# Plasmablastic Lymphoma, a Rare Entity in Bone Marrow with Unusual Immunophenotype and Challenging Differential Diagnosis

**DOI:** 10.1155/2019/1586328

**Published:** 2019-09-02

**Authors:** Majd Al Shaarani, Rodney E. Shackelford, Samip R. Master, Glenn M. Mills, Yasir AlZubaidi, Ahmed Mamilly, Eric X. Wei

**Affiliations:** ^1^Department of Pathology and Translational Pathobiology, Louisiana State University Health Sciences Center, Shreveport, LA, USA; ^2^Department of Hematology and Oncology, Louisiana State University Health Sciences Center, Shreveport, LA, USA; ^3^Department of Radiology, Louisiana State University Health Sciences Center, Shreveport, LA, USA

## Abstract

Plasmablastic lymphoma (PBL) is an aggressive malignancy that usually occurs in the setting of immunosuppression. The immunohistochemical profile of PBL is that of terminally differentiated B lymphocytes. CD138, CD38, and MUM1 are usually immunopositive. However, pan B-cell markers such as CD20 and PAX-5 are usually negative. *MYC* rearrangement is the most commonly encountered genetic alteration, with immunoglobulin (*IG*), especially immunoglobulin heavy (*IGH*) chain, being the most frequent partner. We report a case of PBL in a 48-year-old human immunodeficiency virus- (HIV-) positive male who was admitted to the hospital with signs and symptoms suspicious for tumor lysis syndrome. Bone marrow examination revealed hypercellular marrow with trilineage hypoplasia and sheets of intermediate to large neoplastic cells with basophilic vacuolated cytoplasm comprising the majority of cellular elements of the bone marrow. The neoplastic cells were negative for conventional B-cell, T-cell, plasma cell, and myeloid markers, while flow cytometric analysis revealed an abnormal CD45-dim population that was partially weakly positive for CD71 and CD79b. The diagnosis was initially thought to be a high-grade primitive hematopoietic neoplasm, possibly an acute undifferentiated leukemia. BOB-1, however, was immunopositive in the neoplastic cells, confirming its B-cell origin. MYC was positive by immunohistochemistry and break-apart FISH, as were CD45, MUM-1, and EMA immunostains. There was immunoglobulin kappa (*IGK*) light chain gene rearrangement by polymerase chain reaction (PCR). Additionally, Epstein–Barr virus- (EBV–) encoded small RNAs (EBER) were positive by in situ hybridization (ISH). The tumor proliferation index by Ki-67 immunostaining approached 95%. Although the tumor cells were negative for CD38 and CD138, the diagnosis of PBL was still rendered. We recommend using a broad spectrum of B-cell markers, including BOB-1 and OCT-2, in such challenging cases of B-cell lymphomas with no expression of conventional B-cell markers. We also emphasize that the negative CD38 and CD138 should not exclude PBL from the differential diagnosis.

## 1. Introduction

Plasmablastic lymphoma (PBL) is an aggressive postgerminal center, terminally differentiated B-cell malignancy that usually occurs in the setting of immunosuppression. Initially, PBL was considered a subtype of diffuse large B-cell lymphoma arising in the oral cavity of human immunodeficiency virus- (HIV–) positive patients, as was first described by Delecluse et al. [1–3]. It was later categorized by the WHO as a separate entity in 2008, and accumulative data have demonstrated cases at other anatomical sites and in immunocompetent patients [4]. Although the majority of cases are associated with HIV infection, other causes of immunosuppression such as posttransplantation can also be associated with this lymphoma. About 35% of the patients, however, are actually immunocompetent, indicating that PBL is not confined to the immunosuppressed status. In the majority of cases, the disease arises in the oral cavity of HIV-infected patients. However, other sites are also reported including the gastrointestinal (GI) system, soft tissue, skin, and much less frequently the lungs, bone, bone marrow, and central nervous system (CNS) [5, 6]. The immunohistochemical profile of PBL is that of terminally differentiated B lymphocytes or plasma cells, where CD138, CD38, and MUM1 are usually positive and pan B-cell markers including CD20 and PAX-5 are frequently negative. *MYC* rearrangements are common, often translocated with the immunoglobulin (*IG*) heavy chains (*IGH*), as the most commonly encountered genetic alteration [3, 7]. The rarity of such cases, the difficulty in making the diagnosis, and the very poor prognosis all make PBL a clinicopathological challenge [4].

## 2. Clinical History

**Figure 1:**
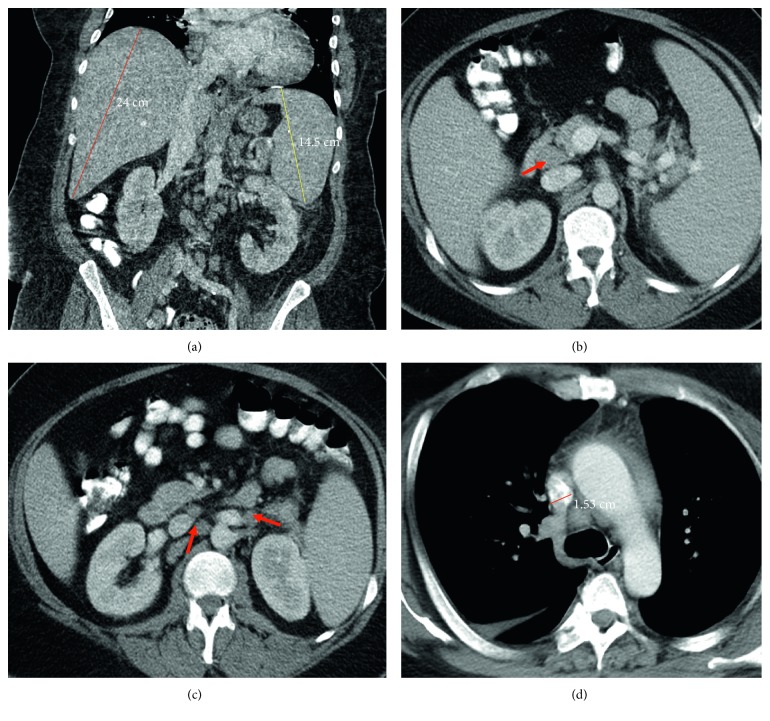
The abdomen and thoracic CT scan. (a) Coronal contrast-enhanced CT image of the abdomen with hepatosplenomegaly: the craniocaudal diameter of the right liver lobe is 24 cm (normal 12–17 cm); the craniocaudal diameter of the spleen is 14.5 cm (normal up to 13 cm). (b) Enlarged mesenteric lymph nodes (red arrow) measure 1.6 cm in short axis. (c) Para-aortic lymph nodes (red arrows) measure about 1.4 and 1.3 cm in short axis. (d) Lymph node in mediastinal station 4R measures about 1.5 cm in short axis.

The patient was a 48-year-old male with a past medical history of HIV, presenting with a historically lowest CD4+ T helper cell count at 73/*µ*L and 190/*µ*L during admission. The patient's past medical history also included HIV-associated immune complex-mediated proliferative glomerulonephritis, chronic kidney disease with multiple episodes of acute kidney injury, electrolyte abnormalities, myocardial infarction, congestive heart failure (ejection fraction of 15–20% two years prior to his most recent admission and then improved to 50–55%), hypertension, diabetes mellitus, anemia, thrombocytopenia, benign prostatic hyperplasia, and major depressive disorder with psychosis and suicidal ideation. He had previously been admitted on several occasions with chief complaints of shortness of breath, hematuria, electrolyte abnormalities, and psychiatric disorders. He eventually developed a subdural hemorrhage confirmed by magnetic resonance imaging (MRI), lactic acidosis, pancytopenia, hypercalcemia, and acute kidney injury (creatinine level of 3.4 mg/dL from 0.93 mg/dL). He reported having progressive dyspnea on exertion over the past few months, along with a chronic cough, productive of green to gray sputum, night sweats, and weight loss of 30 pounds. He denied, however, any hemoptysis, chest pain, paroxysmal nocturnal dyspnea, orthopnea, fever, or chills. He was transferred to the medical intensive care unit and was treated with hydration. His serum calcium level was 13.4 mg/dL, with lactate dehydrogenase levels more than 4000 U/L, and uric acid levels at 26.9 mg/dL. These findings were concerning for tumor lysis syndrome. Chest, abdomen, and pelvic computed tomography (CT) scans revealed hepatosplenomegaly (Figure 1(a)) and retroperitoneal, pelvic, and paracardial lymphadenopathy, with the largest lymph node measuring 2.4 × 1.4 cm (Figures 1(b)–1(d)). A bone marrow biopsy was performed. Initially, it was thought to be a high-grade primitive hematopoietic neoplasm, with acute undifferentiated leukemia in differential diagnosis. However, a diagnosis of plasmablastic lymphoma was later provided. Before chemotherapy could be initiated, the patient developed severe episodes of subdural hemorrhage, lost brain stem reflexes, and subsequently expired after extubation.

## 3. Pathological Findings

**Figure 2:**
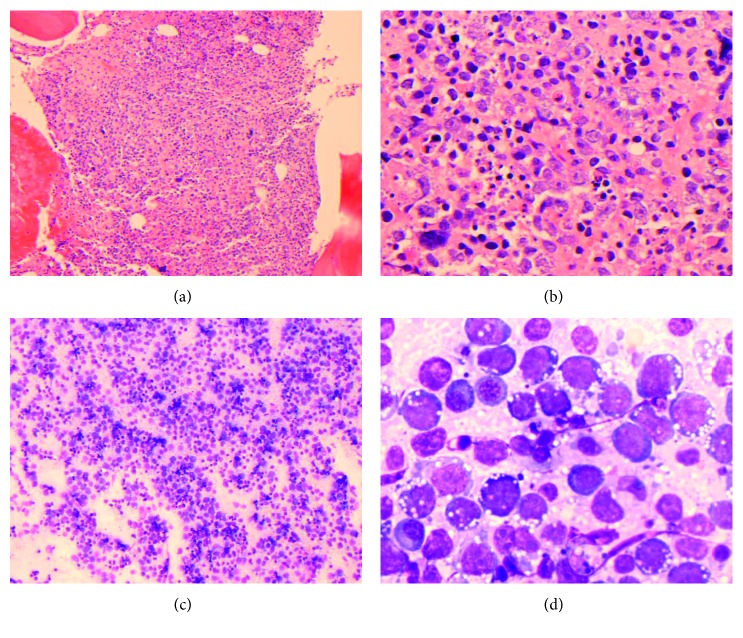
Bone marrow biopsy and aspirate smears show high-grade hematopoietic neoplasm. (a) The neoplasm has a diffuse growth pattern on core biopsy (H&E stain, ×100). (b) The neoplastic cells have irregular nuclear contours, conspicuous nucleoli, and abundant cytoplasm (H and E stain, ×400). (c) Bone marrow aspirate shows discohesive high-grade hematopoietic tumor cells (Wright–Giemsa, ×100). (d) The hematopoietic cells have slightly eccentric nuclei, prominent nucleoli, and deep blue vacuolated cytoplasm. Occasional plasma cells are seen in the background (Wright–Giemsa, ×600).

**Figure 3:**
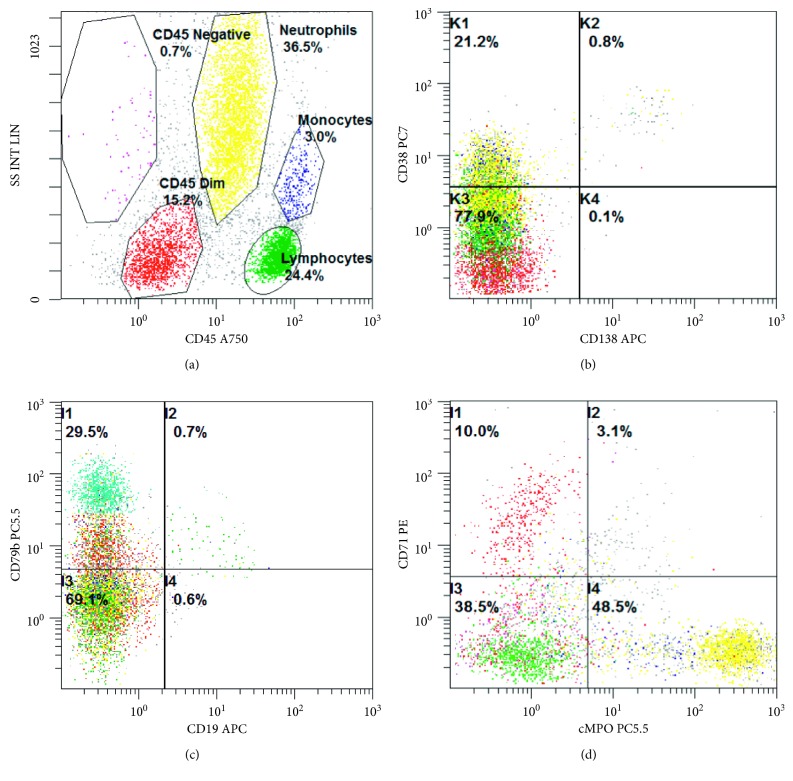
Representative flow cytometric histograms of bone marrow aspirate. (a) There is a CD45-dim population of abnormal cells with low-side scatter (painted red), comprising about 15% of total cells. (b) The cells of interest are negative for CD38 and CD138. (c) These cells are negative for CD19, but partially positive for CD79b, with a subset moderately positive for CD79b (painted blue). (d) These cells are positive for CD71 and negative for cytoplasmic MPO.

**Figure 4:**
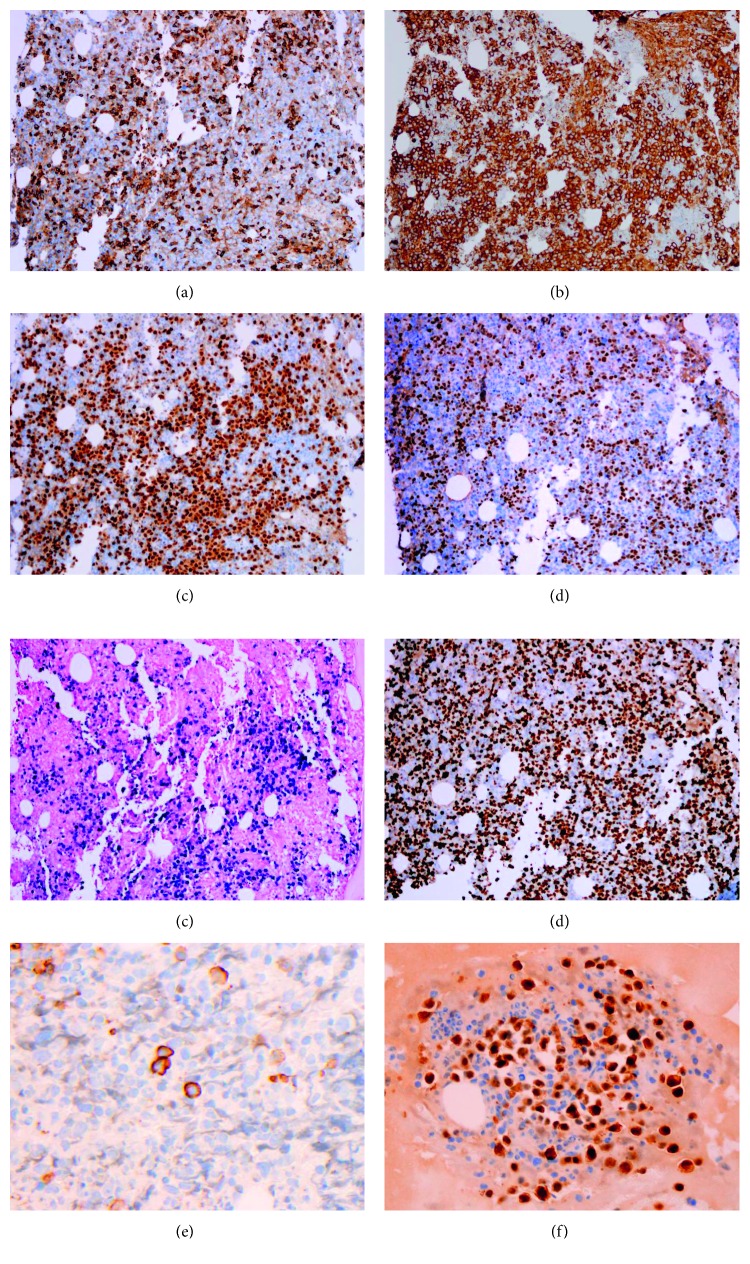
Immunohistochemistry and in situ hybridization study of the high-grade hematopoietic neoplasm. (a) CD45 (×100); (b) EMA (×100); (c) MUM-1 (×100); (d) MYC (×100); (e) EBER (×100); (f) Ki-67 (×100); (g) CD138 (×400); (h) BOB-1 (×200).

**Figure 5:**
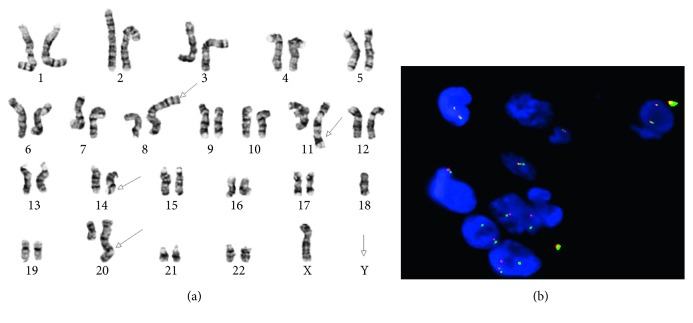
Cytogenetic analysis and FISH study of bone marrow aspirate. (a) The lymphoma cells exhibit complex karyotype, and a representative karyotype shows + der(1), t(1;8)(p13;p12), dup(11)(q13), add(14)(q32), and der(20)t(1;20)(q13.2;q21). (b) *MYC* break-apart FISH is positive for *MYC* gene rearrangement using a locus-specific probe for the 8q24/*MYC* region.

The bone marrow core biopsy and aspirate revealed a hypercellular marrow for the patient's age, at 90% cellularity, with trilineage hypoplasia and slight myelodysplastic changes. The neoplastic cells comprised approximately 75% of the cellular marrow and were arranged in clusters and sheets from intermediate to large cells (Figure 2(a)), with prominent nucleoli and irregular nuclear contours (Figure 2(b)). The bone marrow aspirate smears revealed discohesive neoplastic cells (Figure 2(c)), with a slightly open chromatin and basophilic vacuolated cytoplasm (Figure 2(d)). Flow cytometric analysis showed a distinct CD45-dim cell population, comprising about 15% of the total cells, which were partially positive for CD71 and CD79b and negative for CD34, CD38, CD138, CD117, myeloperoxidase (MPO), and all other B-cell and T-cell markers (Figures 3(a)–3(d)). Immunohistochemically, these tumor cells were positive for CD45 (dim partial), EMA, MUM-1, *MYC* (50%), and Epstein–Barr virus- (EBV-) encoded small RNAs (EBERs), measured by in situ hybridization (ISH) (Figures 4(a)–4(e)). A high proliferation index at 95% was identified by a Ki-67 immunostain (Figure 4(f)). The tumor cells were negative for ALK-1, BCL-1, BCL-2, BCL-6, CAM5.2, CD3, CD10, CD14, CD20, CD30, CD34, CD56, CD68, CD71, CD79a, CD117, CD138 (Figure 4(g)), E-cadherin, factor VIII, glycophorin, HHV-8, lysozyme, MPO, PAX-5, and TdT. There were virtually no normal B-cells by the CD20 and PAX-5 immunostains. The CD3 immunostain identified reactive T-cells. CD79a and CD138 demonstrated background plasma cells, which were polyclonal by kappa and lambda light chain ISH. It was initially considered a high-grade primitive hematopoietic neoplasm, with acute undifferentiated leukemia in the differential diagnosis. Additional immunostains revealed that the neoplastic cells were positive for BOB-1 (Figure 4(h)) and negative for OCT-2. Cytogenetic studies revealed a complicated karyotype 43∼45,X,−Y, der(8)t(1;8)(q12;p22), dup(11)(q13q31), dup(14)(q24q32),−18,der(20)t(1;20)(q12;q13.3)[cp19]/46,XY [1] with a representative karyotype illustrated (Figure 5(a)). Seventeen of the twenty cells were absent for the Y chromosome. Ten cells had this abnormality and a loss of chromosome 18, with a number of unbalanced translocations and duplications, composing a highly complex karyotype carrying a poor prognosis. Two instances of unbalanced 1q translocations led to a quadrupling of this chromosomal arm. The distal portion of 14q was duplicated, as was practically the whole of 11q. Chromosome 18 was present in one copy. None of these abnormalities is known to be characteristic of lymphomas and likely reflect a general chromosomal instability. *MYC* break-apart fluorescent in situ hybridization (FISH) using a locus specific probe for the 8q24/*MYC* region was performed at our institution and was positive for *MYC* gene rearrangement in 9% (9/100) of the cells analyzed with adequate controls (Figure 5(b)). Additionally, a positive peak was detected in a single polymerase chain reaction (PCR) for IG kappa (*IGK*) V-J gene segments, indicating a clonal immunoglobulin kappa light chain gene rearrangement and likely a B-cell lymphoproliferation. There was no evidence of clonal immunoglobulin heavy chain gene rearrangement. The diagnosis of a CD38-/CD138-negative plasmablastic lymphoma was made based on the clinical features of lymphadenopathy, hepatosplenomegaly, HIV and EBV status, B-cell marker BOB-1, CD45, MUM-1, and EMA expression, a high proliferative index, and *MYC* translocation. With this interesting IHC profile, we think it is highly recommended that using a broad spectrum of B-cell markers, including BOB-1, in such cases where other markers are negative or only focally and weakly positive. We also emphasize that a negative CD38 and CD138 expression pattern in tumor cells should not exclude PBL from the differential diagnosis and clinical management.

## 4. Discussion

### 4.1. Clinical Findings

PBL is a rare yet aggressive postgerminal center B-cell malignancy with morphologic and immunophenotypic features of terminal B-cell or plasma cell differentiation. PBL was first described by Delecluse et al. as a subtype of diffuse large B-cell lymphoma (DLBCL) [1]. In the 2008 WHO classification of tumors of hematopoietic and lymphoid tissues, PBL was described as a separate entity, rather than being included under DLBCL, NOS. This disease has a strong male preponderance with approximately seventy percent of cases occurring in males, and the median age of disease presentation is fifty years old [8]. Although, in the majority of cases, the disease arises in the oral cavity of HIV-infected patients, PBL has been reported in other sites including the GI system, soft tissue, skin, and much less frequently in the lungs, bone, bone marrow, and CNS [2, 4, 8, 9]. Initially, the malignancy was thought to be only associated with immunosuppression, especially HIV infection, but later on it was identified in association with other causes of immunosuppression, including iatrogenic interventions such as transplant and autoimmune disorders, immunosenescence, and in immunocompetent patients [8–12]. HIV-positive or posttransplant patients tend to present at disseminated stage III/IV, including bone marrow involvement at presentation when compared to immunocompetent patients. It is possible that PBL may transform from a prior plasma cell myeloma. PBL may sometimes show paraproteins. The majority of PBL patients present with an intermediate-to-high risk international prognostic index. Our patient was a HIV-positive patient, presenting with pancytopenia, hepatosplenomegaly, general lymphadenopathy, possible tumor lysis syndrome, and a high clinical stage involving bone marrow. He did not have oral cavity involvement. Even though hepatosplenomegaly and general lymphadenopathy may suggest high-grade lymphoma, it may also be due to his HIV infection status. His pancytopenia and tumor lysis syndrome may implicate acute leukemia. Thus, accurate pathological diagnosis is essential for recognition of this disease entity and clinical management.

### 4.2. Histopathology

Morphologically, PBL may show variation depending on the sites and possibly the HIV status [6, 8]. Some of the common features include plasmablastic or immunoblastic morphology and plasmacytic differentiation. Mitoses, apoptosis, tingible body macrophages, and occasionally confluent necrosis are characteristic of different disease variations [4, 8, 13]. In HIV-positive patients and in oral and nasal mucosal areas, PBL usually reveals sheets of monomorphic plasmablastic or immunoblastic cell proliferations, with vesicular chromatin, large round- to oval-shaped nuclei, prominent centrally located nucleoli, and abundant cytoplasm. In HIV-negative patients, and in lymph nodes and extraoral cavities, it exhibits a more apparent plasmacytic differentiation with eccentric nuclei, inconspicuous nucleoli, and paranuclear hofs [6, 8, 14].

### 4.3. Immunophenotype

The immunophenotypic profile of PBL is that of terminally differentiated B lymphocytes. CD138, CD38, VS38c, MUM1, PRDMA, and XBP1 positivity is demonstrated in the majority of the cases. However, pan B-cell markers including CD20 and PAX-5 and leukocyte common antigen CD45 are usually negative or can be weakly positive in a minority of cases. CD79a, EMA, and CD30 are often expressed, as well as cytoplasmic immunoglobulin, commonly IgG, frequently with either kappa or lambda light chain restriction. CD4, CD10, CD43, CD45RO, and CD56 have been reported to be expressed in a subset of cases. BCL-1, BCL-2, and BCL-6 expression is usually absent. Ki-67 is usually diffusely positive, typically above 90% [2, 4, 10, 13, 15]. Although the majority of the cases are CD138- and CD38-positive, the diagnosis must not be overlooked if these stains are negative, such as in our case. In a clinical study of 60 Chinese PBL cases, it was found about 12% and 16% of cases were negative for CD38 and CD138, respectively [10]. Dittus and Sarosiek have recently reported an interesting case of PBL in the bone marrow that was positive for CD45, CD79a, CD117, and lambda immunoglobulin light chain but negative for CD38, CD138, PAX-5, and MUM1 [16]. Among the 12 PBL cases described by Teruya-Felstein et al., only one case of a 28-year-old HIV +ve male patient was negative for CD20, CD45, CD79a, CD138, and p63, with the bone and rectum involvement [5]. No other confirmatory B-cell markers were performed. Due to the similar immunohistochemical profile and anatomical locations between our case and reported cases in the literature, it is possible this lymphoma may exhibit different tumor markers when in different anatomical structures. The rarity of such cases and the lack of sufficient data prevent such generalizations. Rare types of B-cell lymphomas may not express conventional B-cell markers, and accurate diagnosis of such cases can be challenging. In an analysis among 34 cases of B-cell lymphomas with no expression of typical B-cell lymphomas, it was found that, in 13 such PBL cases, 9 cases expressed OCT-2, 10 cases had BOB-1 expression, and when combined together, 11 cases were positive for either OCT-2 or BOB-1 [17]. Compared to CD20, CD79a, and PAX-5, which on rare occasions can be found expressed in T-cell lymphomas, acute myeloid leukemia, or nonhematopoietic tumors, OCT-2 and BOB-1 are very sensitive and specific for confirmation of B-cell lymphoma or plasma cell neoplasm that are negative or equivocal for conventional B-cell antigen expression [17]. CD71, a transferrin receptor, is present on actively proliferative cells. It was partially positive in PBL tumor cells in our case by flow cytometry but was negative in bone marrow core biopsy by immunohistochemistry. CD71 expression has been detected by flow cytometry in B-cell lymphomas and acute leukemia [18, 19]. Although weak CD71 expression has been found in DLBCL and subset of acute leukemias, it is selectively expressed at high levels in early and late erythroid precursors and can be used as a lineage-specific marker for erythroid cells in bone marrow by immunohistochemistry [20]. Thus, CD71 partial expression in our PBL case by flow cytometry is nonspecific, and negative CD71 immunohistochemical stain confirms that tumor cells are not in erythroid lineage.

### 4.4. Cytogenetic and Molecular Studies

Our patient showed a complex karyotype with positive *MYC* break-apart FISH results. The dissociation between the karyotype and FISH data may be possibly due to a cryptic *MYC* translocation with an unknown partner gene, undetected by conventional cytogenetics. *MYC* gene rearrangements are the most commonly encountered PBL genetic alteration, with *IG*, particularly *IGH*, being the most frequent partner. These alterations are usually associated with several other complex heterogeneous cytogenetic alterations [3, 7, 8, 10, 21, 22]. Although the causality between EBV infection and *MYC* rearrangements has not been established, *MYC* alterations were more frequently detected when EBV infection was confirmed. Furthermore, Morcsio et al. have shown that EBV was detected in about 75% of AIDS patients' tumor cells but dropped down to 67% in transplant patients and further down to 50% in immunocompetent patients [11]. More studies are needed to clearly demonstrate the causative mechanism, if one actually exists. Al-Malki et al. have demonstrated that the presence of *MYC* gene rearrangement and HIV negativity were adverse prognostic factors [21]. In their review of 60 non-HIV-related PBL cases from Chinese patients, Han et al. found that *IGH* rearrangement was the most frequent alteration accounting for 33.3% of all 14 different types of genetic alterations they found. They also reported, for the first time, the presence of *PML/RARA* fusion in PBL [10].

### 4.5. Differential Diagnosis

The diagnosis of this rare entity depends not only on the histopathological features, but also on the integration of the clinical history, radiologic studies, and laboratory tests. The differential diagnosis encompasses lymphoid tumors with plasmacytic features, including plasmablastic myeloma, ALK-positive diffuse large B-cell lymphoma, Burkitt's lymphoma with plasmacytoid differentiation, immunoblastic DLBCL, and primary effusion lymphoma (PEL) [8, 13, 15]. Clinicopathological correlation appears to be most important factor when trying to differentiate PBL from a plasmablastic myeloma that shows an almost identical histopathological and immunophenotypical profile and is one of the most challenging differential diagnoses. The clinical and laboratory picture of plasmablastic myeloma includes serum and urine paraproteins, hypercalcemia, bone lytic lesions, anemia, and bone marrow involvement. In PBL, on the contrary, positive EBV staining in the tumor cells in the setting of immunosuppression, along with a high proliferative index, and *MYC* translocation supports the diagnosis [8, 23]. The presence of bone lytic lesions, however, should not be used to rule out PBL [16]. Another important diagnosis with similar morphology and immunophenotypical profile to be considered is ALK-positive large B-cell lymphoma [24]. ALK positivity with granular cytoplasmic staining and a t(2;17)(p23;q23) translocation in ALK-positive large B-cell lymphoma help differentiate these two entities [8, 23, 24]. PEL should also be in the differential diagnosis. In patients who are HIV-positive with the solid variant of PEL, differentiating these two entities can be a challenge. HHV-8 positivity is necessary to make the diagnosis for PEL [23, 25]. The expression of pan B-cell antigens (CD20, CD19, and PAX-5) in immunoblastic DLBCL and Burkitt's lymphoma helps differentiate from PBL that is only rarely positive for these markers [1, 8, 10].

### 4.6. Therapy and Prognosis

PBL is a real therapeutic challenge with a very aggressive course. The median overall survival ranges variably between different studies, but generally falls between 11 and 15 months in most cases [22, 26]. The prognostic factors are variable. There appears to be a correlation between survival and HIV status, with a better prognosis when the patient's HIV status is positive. This finding might stem from the administration of highly active antiretroviral therapy (HAART) that generally enhances the immunity in these patients [14]. Radiotherapy and surgery do not appear to have significant effects on patient survival. Currently, there is no consensus for a first-line medication regimen. Although CHOP (cyclophosphamide, doxorubicin, vincristine, and prednisone) and CHOP-like regimens are considered inadequate, other more aggressive regimens like CODOX-M/IVAC (cyclophosphamide, vincristine, doxorubicin, and high-dose methotrexate/ifosfamide, etoposide, and cytarabine), EPOCH (etoposide, cyclophosphamide, doxorubicin, vincristine, and prednisone), or hyper-CVAD (hyperfractionated cyclophosphamide, doxorubicin, vincristine, and dexamethasone) alternating with methotrexate and cytarabine do not confer better results [13, 26]. Bortezomib, especially in combination with either CHOP or EPOCH, has shown a durable remission in few case reports. These reports, however, are limited regarding the numbers of patients and the short follow-up times [26]. A review of the literature shows that brentuximab vedotin, an anti-CD30 antibody, was reported to have been used in only two patients. Either solely [27] or in combination with lenalidomide [26], brentuximab vedotin has demonstrated a very rapid response with significant reduction in the tumor sizes [26, 27]. Lenalidomide has also shown similar results in a few reported cases [26, 28, 29]. In a study of 82 PBL patients, Laurent et al. showed immune checkpoint expression (PD1/PD-L1) in the tumor microenvironment in the majority of cases and about 25% of the tumor cells expressed PD-L1. They could not find any correlation with survival, but they showed a rationale for testing anti-PD-L1 antibodies for the treatment of PBL [30].
